# Corrosion assessment in aluminum pipe based on nonlinear ultrasonic technique using macro fiber composite transducers

**DOI:** 10.1371/journal.pone.0353469

**Published:** 2026-07-10

**Authors:** Rong Wang, Hanqi Zhang, Heng Chen, Yahong Wu, Ke Xiong, Qi Wu, Liqing Zou

**Affiliations:** 1 College of Traffic Engineering, Nanjing University of Industry Technology, Nanjing, China; 2 State Key Laboratory of Mechanics and Control for Aerospace Structures, Nanjing University of Aeronautics and Astronautics, Nanjing, China; 3 School of Mechanical and Electronic Engineering, Nanjing Forestry University, Nanjing, China; Chongqing Medical University, CHINA

## Abstract

Pipe corrosion, specifically pitting corrosion, is the main cause of destructive pipe leakage, driven by the harsh working environment of liquid and gas transportation. Therefore, detecting pitting corrosion is essential for ensuring the safe operation of metal pipes. This study investigates a nonlinear ultrasonic technique using macro fiber composite transducers, aiming to assess pitting corrosion in metal pipes at an early stage, with a focus on characterizing the influence of temperature on the ultrasonic nonlinearity. Macro fiber composite transducers with flexibility and high ultrasonic performance were used to actuate and detect ultrasonic guided waves propagating in pipes with curved surfaces. Considering the multi-mode propagation characteristics, the 1.4 MHz second-harmonic ultrasonic component generated by the nonlinear interaction between ultrasonic guided waves and pitting corrosion was extracted. Repeated experiments revealed that both the second-harmonic amplitude and relative nonlinear parameter exhibited a monotonic increase with the number of cycles and area of pitting corrosion. For comparison with the nonlinear results, statistical metrics including the mean slopes, coefficient of determination, and relative standard deviation of the linear fitting parameter were determined alongside the linear ultrasonic experiments. These results indicate that, despite some inherent data variability, the proposed nonlinear ultrasonic technique exhibits comparatively better sensitivity, goodness-of-fit and repeatability than linear ultrasonic methods for identifying pitting corrosion. Thus, the proposed nonlinear ultrasonic technique using macro fiber composites offers a promising complementary alternative for early corrosion assessment in metal pipes.

## Introduction

Metal pipes are critical for transporting natural gas, fuel oil, chemicals, and water because of their high specific strength and overall reliability [1, 2]. However, metal pipes are susceptible to corrosion damage, which can lead to structural failure during long-term service [3, 4]. Material corrosion can be classified into localized corrosion and general corrosion, with the occurrence of the former being significantly higher. Among the various forms of localized corrosion, pitting corrosion is one of the most destructive and severe in pipe structures [5]. Moreover, pitting corrosion promotes secondary degradation mechanisms such as intergranular corrosion, stress corrosion cracking, and corrosion fatigue. Pitting corrosion often causes leakage accidents or even explosions, not only resulting in substantial economic losses but also posing a major threat to human safety [6]. Therefore, developing effective techniques for detecting pitting corrosion is essential.

Currently, technologies such as laser scanning, microwave inspection, pulsed eddy current, electromagnetic induction thermography, and ultrasonic detection are used for damage detection in pipe structures. Laser scanning provides high resolution and rapid detection speed but incurs high equipment costs [7]. Microwave inspection cannot detect internal damage within metallic materials [8]. While pulsed eddy current and electromagnetic induction thermography technologies overcome some of these drawbacks and can detect microdamage [9, 10], they—along with laser scanning and microwave inspection—are typically deployed as offline nondestructive testing (NDT) techniques. Consequently, they are ill-suited for real-time or online continuous monitoring of corrosion damage. These offline methods often require operational downtime and are generally limited to scheduled maintenance intervals. As a result, they cannot capture the dynamic propagation of damage during the operational life of structures, thereby increasing the risk of catastrophic failure.

In practical applications, there is a high demand for effective and efficient pipe condition assessment. Compared with other detection methods, ultrasonic detection offers distinct advantages, including high efficiency, low cost, long-range capability and adaptability to pipes of various sizes and thicknesses [11, 12]. Traditional ultrasonic methods typically evaluate damage by analyzing changes in fundamental wave parameters, such as amplitude, arrival time, attenuation, and velocity dispersion. However, parameters like arrival time are not uniquely associated with damage and can be significantly influenced by environmental factors including temperature variations, stress states, and material anisotropy. While these methods detect distributed damage such as porosity or diffuse cracking through changes in attenuation or dispersion, they are generally less sensitive than nonlinear ultrasonic methods to microdamage because the ultrasonic wavelength exceeds the damage scale [13]. Consequently, nonlinear ultrasonic methods have recently been proposed to address these limitations. These techniques can detect microdamage by analyzing the generation of higher harmonic components and side-lobe frequencies in the frequency domain [14]. Unlike conventional linear ultrasonic detection, nonlinear ultrasonic methods are highly sensitive to microdamage because they evaluate material degradation through the nonlinear effects generated by wave–damage interactions [15]. A comprehensive comparison of the advantages and drawbacks of various detection technologies (Table 1).

**Table 1:** Advantages and drawbacks of the detection techniques.

Detection technology	Advantages	Drawbacks
laser scanning	high resolution	offline, high cost
microwave inspection	centimeter-level depth detection	offline, only applied to nonmetal/composites materials
pulsed eddy current	deep damage detection	offline, limited to localized damage
electromagnetic induction thermography	rapid detection	offline, high cost
linear ultrasonic detection	long-distance detection	online, misdetection of geometrical abrupt structures
nonlinear ultrasonic detection	long-distance detection	online, complicated analysis process

Early damage in pipes can be successfully detected by analyzing nonlinear ultrasonic phenomena, including higher harmonic generation, frequency mixing responses, and shifts in harmonic frequency [16, 17]. Higher harmonic generation provides high sensitivity for identifying microdamage by detecting the damage-induced material nonlinearity, thereby improving the identification of early-stage damage [18]. To mitigate the risk of false positives, potential sources of nonlinearity including instrumentation, coupling variability, and boundary conditions must be carefully controlled. Consequently, higher harmonic generation is considered the most classical nonlinear phenomenon, making it highly suitable for practical applications. For example, Hong et al. [19] reported that second- and third-order nonlinear coefficients increased with delamination damage in lined anticorrosion pipes. Ehrlich et al. [20] developed ultrasonic technology to detect nonlinearity parameters in a welded steel pipe, reporting that high levels of nonlinearity occurred in the heat-affected zone as creep damage accumulated. Hong et al. [21] detected stress corrosion cracking in stainless steel rods using nonlinear resonant ultrasound spectroscopy under specific stress and corrosion conditions, finding a general exponential relationship between the corrosion exposure time and the nonlinear parameter. Zhong et al. [22] utilized the nonlinear characteristics of ultrasonic waves to detect the intergranular corrosion of steel tubes and reported that the nonlinear coefficient generally increased with the degree of intergranular corrosion damage. Although these studies have validated the use of nonlinear ultrasound for detecting general pipeline corrosion, research specifically addressing the pitting corrosion of metal pipes remains limited. This limitation is primarily due to the minute size of pitted area, which makes accurate detection highly challenging.

Corrosion assessment is further complicated by the geometric shape and operating conditions of the pipes. Unlike large-diameter pipes, where rigid transducers suffice and guided waves can often be approximated as plate-like waves, small-diameter pipes exhibit complex cylindrical dispersion and severe curvature. This curvature renders conventional rigid transducers ineffective due to substantial acoustic energy loss. Moreover, traditional lead–zirconate–titanate (PZT) ultrasonic transducers are inherently rigid with flat surfaces, making acoustic coupling challenging on highly curved small-diameter pipe geometries. This lack of surface contact often leads to large acoustic energy loss at the interface. Additionally, the pipe always operates in a humid environment, which affects the corrosion resistance of the transducer. To mitigate these shortcomings, researchers have recently proposed macro fiber composite (MFC) transducers. MFCs are a new type of functional composite material composed of a polymer film embedded with ceramic fibers, exhibiting a strong piezoelectric effect. Compared to traditional PZT transducers, MFCs offer significantly superior flexibility, driving ability, and corrosion resistance [23], making them a highly promising alternative for the ultrasonic detection of pipe structures.

The objective of this paper is to investigate the interaction between ultrasonic guided waves and pitting corrosion in small-diameter pipes using MFC transducers. Specifically, this study establishes the relationship between ultrasonic nonlinear characteristics and the number of cycles and area of pitting corrosion. Achieving effective detection of localized corrosion damage enables better differentiation between localized and global damage effects on ultrasonic characteristics. In this study, a nonlinear ultrasonic method using flexible MFC transducers is proposed to assess the number of cycles and area of pitting corrosion in aluminum pipes. Compared to conventional linear ultrasonic detection, this proposed technique provides better sensitivity, goodness of fit and repeatability. This paper represents the first application of higher-order harmonic nonlinear ultrasonic technology to detect pitting corrosion in small-diameter pipelines. Moreover, a comparative analysis with traditional linear ultrasonics is conducted to verify the reliability and accuracy of the proposed nonlinear ultrasonics in these cases. This paper is organized as follows: After the Introduction section, the principles and parameters of nonlinear ultrasonic detection based on higher harmonic generation are explained in the Principles of nonlinear ultrasonic detection section. An ultrasonic experimental setup for the MFC and pitting corrosion experiments is described in the Pipe experiment setup section. In the Nonlinear ultrasonic results section, the relationships between pitting corrosion and the ultrasonic amplitude, second-harmonic amplitude and nonlinear parameter are analyzed. Finally, conclusions are presented in the Conclusion section.

## Principles of nonlinear ultrasonic detection

**Fig 1:**
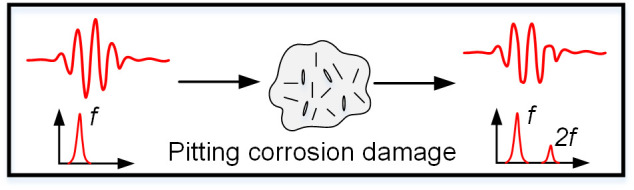
Schematic diagram of nonlinear ultrasonics.


Higher harmonic generation stems from the interaction between ultrasonic guided waves and structural damage within a material. When a localized area of an aluminum pipe comes into contact with an electrolyte solution, numerous small-scale craters and microcracks form in disordered clustered [24]. This damage interacts with propagating ultrasonic guided wave (Fig 1). As an ultrasonic guided wave propagates through a microcrack, its compressive components close the crack, allowing the wave to penetrate the crack interface. Conversely, the tensile components open the crack and block the propagation of the guided wave. The continuous opening/closing motion of the crack distorts the wave, generating contact acoustic nonlinearity (CAN), denoted as β_CAN_ [25, 26]. CAN originates from the nonlinearity of the contact state at solid interfaces, and it is an acoustic nonlinearity caused by the mechanical behaviors of the contact interface, such as elastic contact, microslip, and separation. Furthermore, small-scale craters introduce irregular geometric shapes on the surface or within the structure. These irregularities cause the pipe to exhibit nonlinear behavior during the propagation of ultrasonic guided waves, resulting in geometric nonlinearity β_G_ [27]. Geometric nonlinearity stems from the structural deformation effects where the relationship between displacement and strain is no longer linear. This type of nonlinearity is induced by geometric deformation and remains independent of the linearity or nonlinearity of the material itself. Moreover, small-scale craters induce localized variations in material strength, elasticity, and hardness, generating material nonlinearity β_M_ due to altered mechanical behaviors compared to unaffected areas [28]. Material nonlinearity originates from the intrinsic nonlinear constitutive relationship of the material; that is, stress and strain do not conform to linear Hooke’s law. This nonlinearity is an intrinsic characteristic of the material itself. The total ultrasonic nonlinearity β induced by pitting corrosion can be expressed as follows:

$$\begin{array}{c} \beta =\beta_{CAN}+\beta_{M}+\beta_{G} \end{array}$$ (1)

In a solid material, the material and geometric nonlinearity can be depicted using the nonlinear stress‒strain relation with a second-order approximation according to the nonlinear Hook’s law [29] expressed as follows:


$$\begin{array}{c} \sigma =E\varepsilon +E\beta \varepsilon^{\text{2}} \end{array}$$ (2)

where *σ* represents the stress, *ε* denotes the strain, and *E* represents the Young’s modulus, and β represents the second-order nonlinear parameter. According to the progressive approximation perturbation theory [30], the approximate solution of the 1D nonlinear longitudinal wave equation yields the fundamental and second-harmonic components. The nonlinear parameter *β* can be expressed as follows [31]:


$$\begin{array}{c} \beta \text{=}\frac{8A_{\text{2}}}{\overset{\text{2}}{\underset{\text{1}}{A}}k^{\text{2}}x} \end{array}$$ (3)

where $A_{\text{1}}$ and $A_{\text{2}}$ represents the amplitude of the fundamental and second-harmonic wave, respectively. When the wavenumber and propagation distance are fixed, the nonlinear parameter can be obtained by calculating the amplitudes of the fundamental and second-harmonic waves. To simplify the calculation process of the nonlinear ultrasonic characteristics, the relative nonlinear parameter $\beta^{\prime }$ is used to represent the ultrasonic nonlinearity instead of β [32]:


$$\begin{array}{c} \beta^{\prime }=\frac{A_{\text{2}}}{\overset{\text{2}}{\underset{\text{1}}{A}}} \end{array}$$ (4)

When ultrasonic guided waves propagate through a medium with microcracks, higher-order harmonic waves are generated because of contact acoustic nonlinearity. The CAN can be simplified as the elastic modulus change at microcrack z_1_, where the stress is expressed as


$$\begin{array}{c} \sigma_{\text{1}}\left(z_{\text{1}}\right)=E_{\text{1}}\left(z_{\text{1}}\right)\varepsilon_{\text{1}}=\left(1-\beta_{CAN}max\left(\frac{\partial u\left(z_{\text{1}}\right)}{\partial z}\right)\right)E_{\text{0}}\frac{\partial u\left(z_{\text{1}}\right)}{\partial z}+\beta E_{\text{0}}{\left(\frac{\partial u\left(z_{\text{1}}\right)}{\partial z}\right)}^{\text{2}} \end{array}$$ (5)

where $E_{\text{0}}$ represents the initial elastic modulus, $E_{\text{1}}$ denotes the average value of the elastic modulus after crack $z_{\text{1}}$ occurs, $u_{\text{1}}$ indicates the displacement at microcrack $z_{\text{1}}$, $\varepsilon_{\text{1}}$ represents the strain at microcrack $z_{\text{1}}$, and $\beta_{CAN}$ denotes the acoustic nonlinear parameter. Through derivation, the second-harmonic wave can be further simplified as follows [33]:


$$\begin{array}{c} A_{\text{2}}=-\frac{\beta_{CAN}ck^{\text{2}}{\left(A_{\text{1}}\right)}^{\text{2}}}{\text{8}}cos(2kz_{\text{1}}-2\omega t) \end{array}$$ (6)

Therefore, $\beta_{CAN}\approx A_{\text{2}}/\overset{\text{2}}{\underset{\text{1}}{A}}$, which can also be expressed as $\beta^{\prime }$. $\beta^{\prime }$is a suitable damage index for identifying nonlinear parameter changes in pipes and, therefore, for analyzing the pitting corrosion state [34].

### Dispersion characteristics in small-diameter pipes

**Fig 2:**
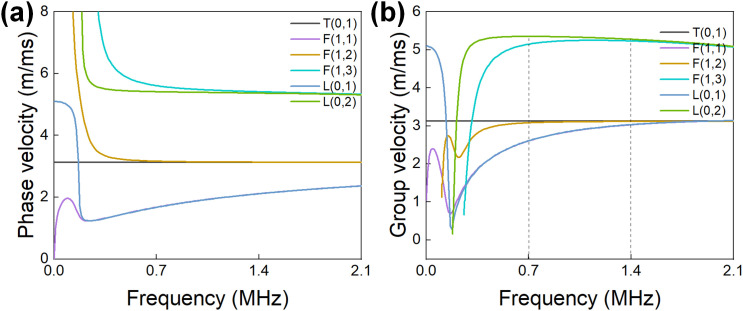
Dispersion curve of aluminum pipe of the outer radius 10 mm with the thickness of 0.5 mm (a) phase velocity; (b) group velocity.

Compared to large-diameter pipelines, small-diameter pipes exhibit more complex ultrasonic guided wave properties and more congested dispersive modes. Theoretical dispersion curves for the aluminum pipe are calculated by the Dispersion Calculator (https://github.com) from 0–2.1 MHz (Fig 2). Longitudinal (L), transverse (T) and flexural (F) modes can all propagate within the 2017 aluminum pipes, whose outer radius is 10 mm and whose wall thickness is 0.5 mm. When the input frequency is 700 kHz, the primary modes L(0, 2) and F(1, 3) modes exhibit very similar velocities, leading to time-domain signal overlap and phase interference. This complex waveform complicates signal interpretation and contributes to the variability observed in the nonlinear ultrasonic detection. In the subsequent experimental sections, this study will distinguish the L(0,2) mode from the F(1,3) mode by utilizing the phase characteristics of the upper and lower signals and optimizing the MFC sensor placement.

## Pipe experiment setup

### Nonlinear ultrasonic experiment

**Fig 3:**
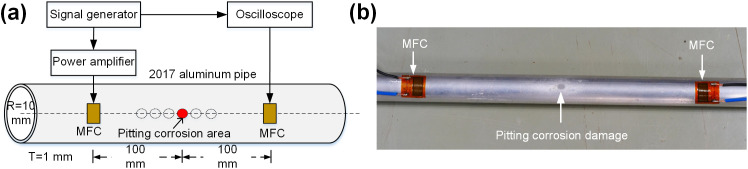
Nonlinear ultrasonic experimental setup: (a) schematic diagram of the experimental system and (b) aluminum pipe with pitting corrosion.

Nonlinear ultrasonic experiments were conducted on 2017 aluminum pipes with lengths, inner radii, and outer radii of 300, 9, and 10 mm, respectively (Fig 3a). The material properties of the aluminum include a Young’s modulus E of 72.4 GPa, a Poisson’s ratio ν of 0.33, and a mass density ρ of 2.79 × 10^3^ kg/m^3^. Due to the small outer radius of aluminum pipes, traditional PZT sensors with rigid surfaces could not be attached to the curved surfaces of pipe structures. Consequently, MFC transducers with good flexibility were used instead of PZTs to detect ultrasonic guided waves. Two MFC transducers (Smart Material Corp., P2 type) had dimensions of 7 × 14 × 0.4 mm (width × length × thickness). These transducers functioned as a sensor and an exciter and were glued 200 mm apart using a cyanoacrylate adhesive (Fig 3b). The MFC was driven by a 10-cycle sine tone-burst signal modulated by a Hamming-window with a center frequency of 0.7 MHz. This frequency was selected because higher input frequency can increase the nonlinear ultrasonic detection sensitivity, and 0.7 MHz represents the operational bandwidth limit of the MFC in excitation mode [35, 36]. The input signal was generated by a function generator (Agilent, 33521A) and amplified to 80 V using a power amplifier (Aigtek, ATA-43151). An oscilloscope (Keysight, DSOX2004A) with a sampling frequency of 10 MHz was used to capture the received waveforms, which were recorded after 8192-fold averaging to minimize noise. All the experiments were conducted in a controlled temperature and humidity environment.

### Corrosion experiment

**Fig 4:**
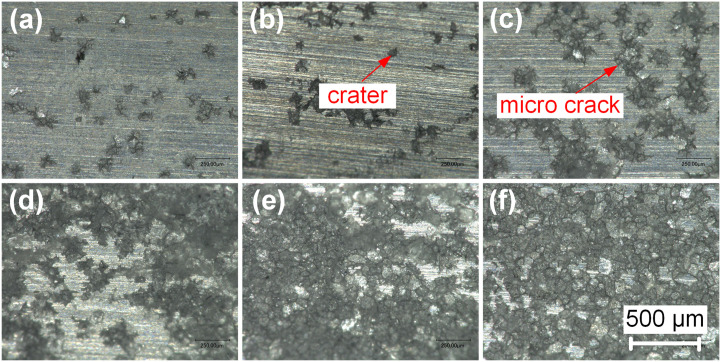
Generated pitting corrosion damage after (a) one, (b) two, (c) three, (d) four, (e) five, and (f) six cycles of hydrochloric acid dropping.

To induce controlled pitting corrosion, 36% dilute hydrochloric acid was applied dropwise to designated points between the MFC transducers on the aluminum pipe using a disposable plastic syringe. The localized corrosion damage area generated on the aluminum pipe after 20 minutes was considered the completion of one pitting corrosion cycle; that is, one corrosion cycle was defined as a single 20-minute exposure to the acid. In practical applications involving metal pipes, pitting corrosion not only accumulates in a single area but also expands along the surface to create pitting corrosion groups [37, 38]. Therefore, two experiments were conducted to investigate the nonlinearity induced by cumulative pitting corrosion damage at a single point and pitting corrosion damage over a relatively large area. The first pitting corrosion experiment was repeated six times at the same center point on three identical pipes, with ultrasonic signals detected after each pitting corrosion cycle. The pitting corrosion area was circular with a diameter of 5 mm (Fig 3b). Micrographs of the corrosion area were obtained using an optical microscope (Nreeohy, S-Y500) (Fig 4), which revealed the presence of disordered clusters of small-scale craters and microcracks. Both the density and dimensions of this damage increased with the number of pitting corrosion cycles [39]. These boundaries of these microcracks and small-scale craters function as critical contact interfaces that exhibit clapping or breathing behavior during ultrasonic guided wave propagation. This mechanism of contact acoustic nonlinearity induces non-sinusoidal distortion of the wave packets and generates higher-order harmonics. In the second pitting corrosion experiment, six similar shaped corrosion areas were created at 5 mm intervals near the central points of pipes, and an ultrasonic signal was detected after each pitting corrosion. The numbers of craters and microcracks increased with increasing pitting corrosion area. Both experiments were repeated three times to ensure the repeatability and statistical reliability of the results.

## Nonlinear ultrasonic results

### Typical ultrasonic guided wave

**Fig 5:**
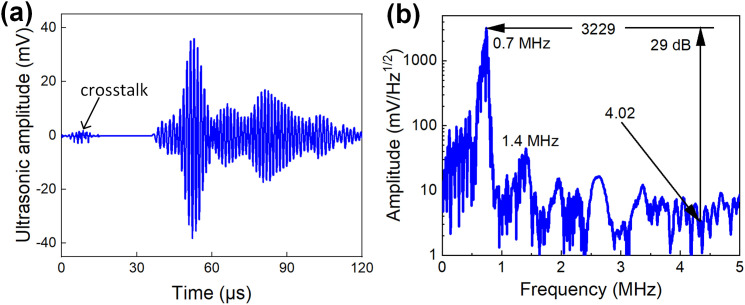
Ultrasonic signal of the MFC: (a) time domain and (b) fast Fourier transform.

**Fig 6:**
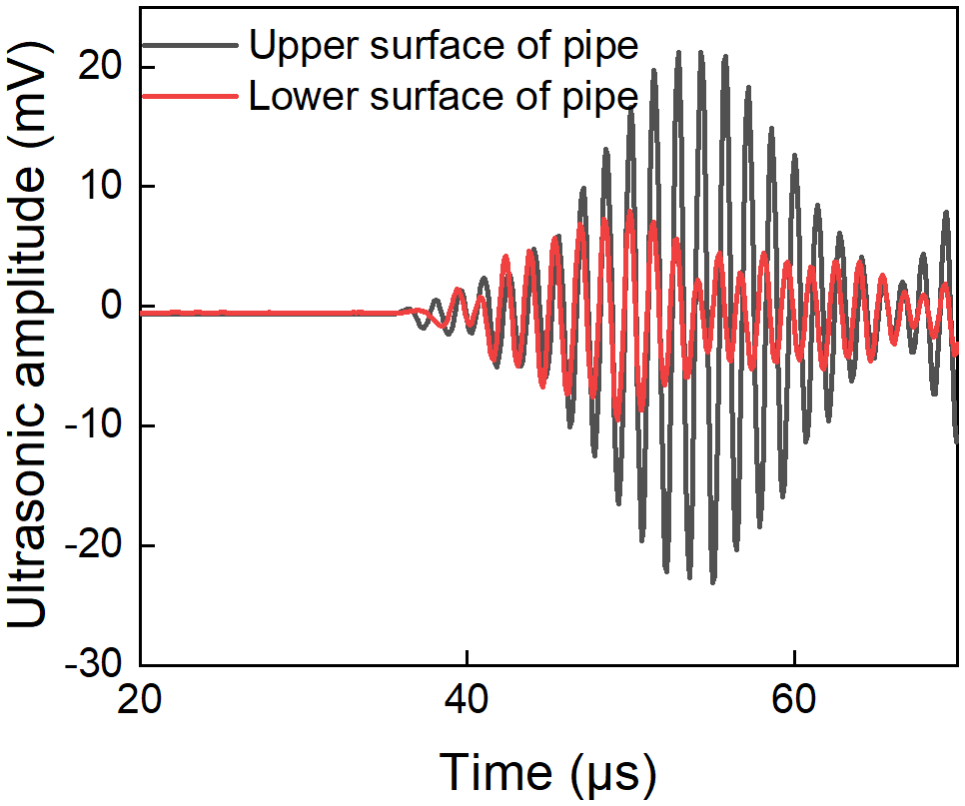
Ultrasonic signals on the upper and lower surfaces of the pipe.

A typical ultrasonic signal is detected by the MFC transducers (Fig 5a). A minor fluctuation observed at approximately 9 μs was identified as crosstalk. With a propagation distance of 200 mm, the signal reached a peak amplitude of 52 mV at an arrival time of 50 μs. This arrival time was consistent with the group velocities of the L(0, 2) and F(1, 3) modes based on dispersion calculations (Fig 2) [16]. The frequency spectrum of the signal was obtained via Fast Fourier Transform (FFT) (Fig 5b). The primary energy was concentrated at 0.7 MHz, matching the excitation signal. A small amount of energy was concentrated at the second-harmonic frequency of 1.4 MHz. The signal-to-noise ratio of the MFC transducer was calculated at 29 dB, indicating high ultrasonic sensitivity in the experimental setup. To ensure the accuracy of the nonlinear ultrasonic analysis, it is critical to address the potential mode mixing between L(0, 2) and F(1, 3) modes, which exhibit similar group velocities at the excitation frequency of 700 kHz. The MFC transducers were specifically aligned and attached along the longitudinal axis of the aluminum pipes, as shown in (Fig 3b). This axial orientation utilizes the d_33_ effect of the MFC to predominantly excite longitudinal strain, thereby maximizing the energy coupled into the symmetric L(0, 2) mode while suppressing flexural modes like F(1, 3). Moreover, ultrasonic signals were collected from both the upper and lower surfaces of the pipe at the same propagation distance (Fig 6). The results show that the ultrasonic guided wave packets on opposite sides of the pipe wall are in-phase, confirming the dominance of the symmetric L(0,2) mode.

**Fig 7:**
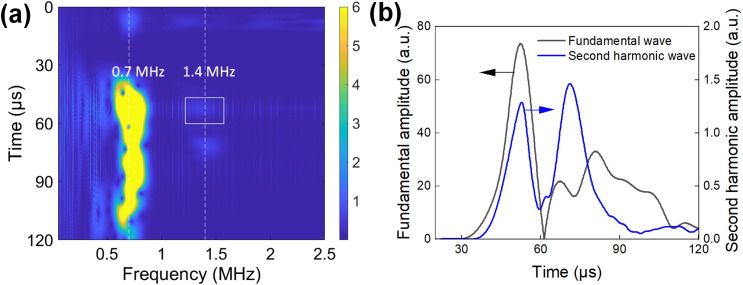
Wavelet transform of the signal detected by MFC transducers: (a) spectrogram in time–frequency domain and (b) $A_{1}$ and $A_{2}$ obtained from amplitude envelopes at the fundamental and harmonic frequencies.

To extract the effective fundamental and harmonic waves, the wavelet transform is preferred over the FFT for amplitude extraction due to its time-frequency resolution. The FFT uses the signal over the entire time window, which makes the results susceptible to boundary reflections and slower modes. In contrast, the wavelet transform enables precise amplitude extraction exactly at the time of the first arrival peak. This temporal precision is essential for effectively extracting the L(0, 2) wave packet and ensuring the accuracy of the relative nonlinear parameter $\beta^{\prime }$. Therefore, a wavelet transform with a mother wavelet “cmor 2-2.5” was used in this study. Cmor 2–2.5 is a complex Morlet wavelet defined by a positive time-decay parameter $F_{b}=\text{2}$ and a positive center frequency $F_{c}=\text{2}.\text{5}$ [40]. The time-frequency spectrogram of the signal after data processing is presented (Fig 7a). Most of the energy was concentrated at approximately 0.7 MHz, and a small amount of energy existed at a frequency component of 1.4 MHz. These two frequency components correspond to the fundamental and harmonic ultrasonic guided waves. The envelopes were then extracted individually using the Hilbert transform (Fig 7b). Substituting the amplitudes selected at the first peaks of each wave, which are denoted as $A_{\text{1}}$ and $A_{\text{2}}$, into Eq 4 yields the relative nonlinear parameter $\beta^{\prime }$. Because the amplitudes of the fundamental and harmonic waves, which were extracted by the wavelet transform, do not overlap with the arrival time window of the crosstalk signals, the crosstalk present in the ultrasonic signals does not affect the experimental results. Although multimodal propagation exists at the excitation frequency, the symmetric placement of the MFC transducers effectively minimizes the excitation of flexural modes such as F(1, 3). Since L(0, 2) remains the fastest propagating mode, extracting $A_{\text{1}}$ and $A_{\text{2}}$ from the first arrival peaks further separates it from any residual slower modes. Furthermore, while damage-induced linear mode conversion may attenuate $A_{\text{1}}$, it cannot generate new frequencies. Therefore, the 1.4 MHz second-harmonic wave is primarily caused by pitting corrosion-induced nonlinearity rather than changes in modal content.

### System linearity validation

**Fig 8:**
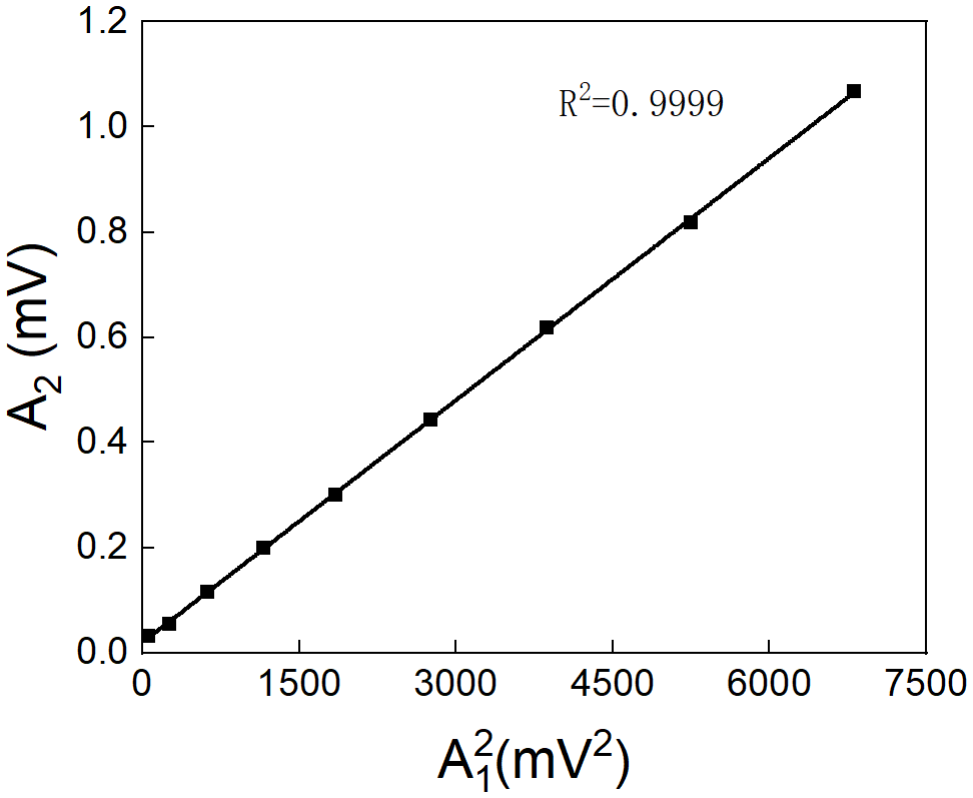
$A_{2}$ versus $\overset{2}{\underset{1}{A}}$ under different excitation voltages.

To verify the transducer’s sensitivity at 1.4 MHz, a system linearity experiment was conducted by changing the excitation voltage from 10 V to 90 V. As previously described, a complex Morlet wavelet transform (cmor 2–2.5) was applied to separate the fundamental and second-harmonic frequency components, followed by a Hilbert transform to obtain the respective signal envelopes. The fundamental and second-harmonic amplitudes $A_{\text{1}}$ and $A_{\text{2}}$ were extracted from the first peaks of each wave envelope. The second-harmonic amplitude $A_{\text{2}}$ increased linearly with the square of the fundamental amplitude $\overset{\text{2}}{\underset{\text{1}}{A}}$, with a coefficient of determination $R^{\text{2}}$ of 0.9999 (Fig 8). The linear relationship follows the equation $A_{\text{2}}=\text{1}.\text{5}\text{3}\times {\text{1}\text{0}}^{-\text{4}}\overset{\text{2}}{\underset{\text{1}}{A}}+\text{0}.\text{0}\text{1}$9. The small positive intercept of 0.019 indicates an inherent nonlinearity caused by the measurement system. However, this constant system nonlinearity does not affect accurate characterization, as the pitting corrosion assessment primarily depends on the relative increase of $\beta^{\prime }$. More importantly, this excellent goodness-of-fit ($R^{\text{2}}=\text{0}.\text{9}\text{9}\text{9}\text{9}$) serves as a critical validation for the sensor's performance at 1.4 MHz. Despite a reduction in absolute sensitivity beyond the nominal bandwidth, this experiment confirms that the sensors maintain a stable and highly linear response.

To evaluate the goodness-of-fit of the regression model, the coefficient of determination ($R^{\text{2}}$) is calculated, and the formula for $R^{\text{2}}$ is defined as follows:


$$\begin{array}{c} R^{\text{2}}=1-\frac{\overset{n}{\underset{i=1}{\sum }}{\left(y_{i}-{\overset{\mathrm{\backslash lower}0.5em\mathrm{\backslash smash}⌢\$}{\}}y}_{i}\right)}^{\text{2}}}{\overset{n}{\underset{i=1}{\sum }}{\left(y_{i}-\overset{―}{y}\right)}^{\text{2}}} \end{array}$$ (7)

where $y_{i}$ represents the observed value of the dependent variable for the *i*-th data point, $\hat{y}$_*i*_ denotes the predicted (fitted) value from the line $\hat{y}=a+bx$ for the i-th data point, $\overset{―}{y}$ indicates the mean of the observed dependent variable, n represents the total number of data points, and ∑ denotes the sum over all n data points. An $R^{\text{2}}$ value closer to 1 indicates a better fit of the experimental data to the applied model.

### Temperature experiments

**Fig 9:**
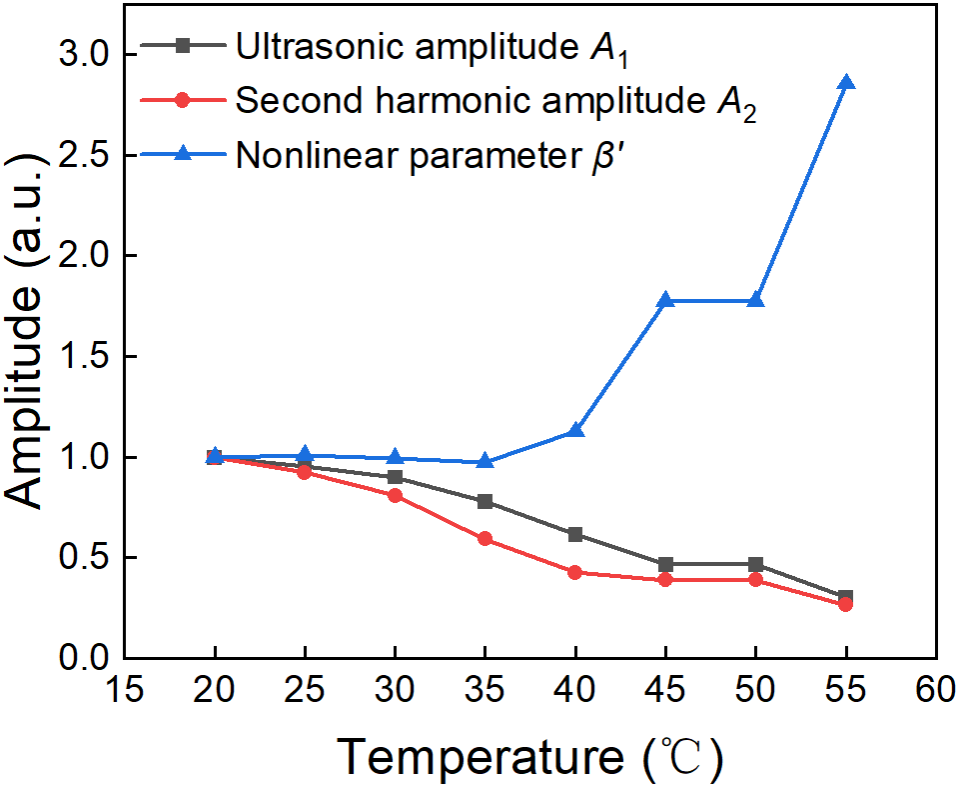
Temperature versus $A_{1}$, $A_{2}$ and $\beta^{\prime }$.

Temperature experiments were performed on an identical pipe within a range of 20 ℃ to 55 ℃ using an oven (MROBO, JYG45), ultrasonic signals were detected at 5 ℃ intervals. The relationships among the linear ultrasonic amplitude $A_{\text{1}}$, second harmonic amplitude $A_{\text{2}}$, and relative nonlinear parameter $\beta^{\prime }$ and temperature are presented (Fig 9). Both $A_{\text{1}}$ and $A_{\text{2}}$ decreased monotonically with increasing temperature, dropping by approximately 70% from 20 °C to 55 °C This significant amplitude reduction resulted primarily from enhanced material damping and internal friction at higher temperatures, which increased ultrasonic attenuation. Furthermore, the weakening of the adhesive layer and changes in transducer sensitivity led to acoustic energy loss. When the temperature increased from 20°C to 35 °C, $\beta^{\prime }$ remains relatively stable with fluctuations limited to roughly 3%. However, $\beta^{\prime }$ began to increase rapidly above 35 °C and reached 2.8 times its baseline value by 55 °C. This two-stage behavior indicated that $\beta^{\prime }$ is stable under small temperature changes but increases rapidly at higher temperatures. This is mainly because the fundamental wave attenuates much faster, leading to a very small $\overset{\text{2}}{\underset{\text{1}}{A}}$ value in Eq 4, alongside temperature-induced changes in the material’s third-order elastic constants. These findings indicate that $A_{\text{1}}$ and $A_{\text{2}}$ are highly susceptible to temperature variations across the entire range. In contrast, $\beta^{\prime }$ is less affected by temperature when it is below 35 °C. Consequently, subsequent ultrasonic experiments were maintained at a controlled room temperature of approximately 25 °C to enhance detection accuracy.

### Assessment of pitting corrosion cycles

**Fig 10:**
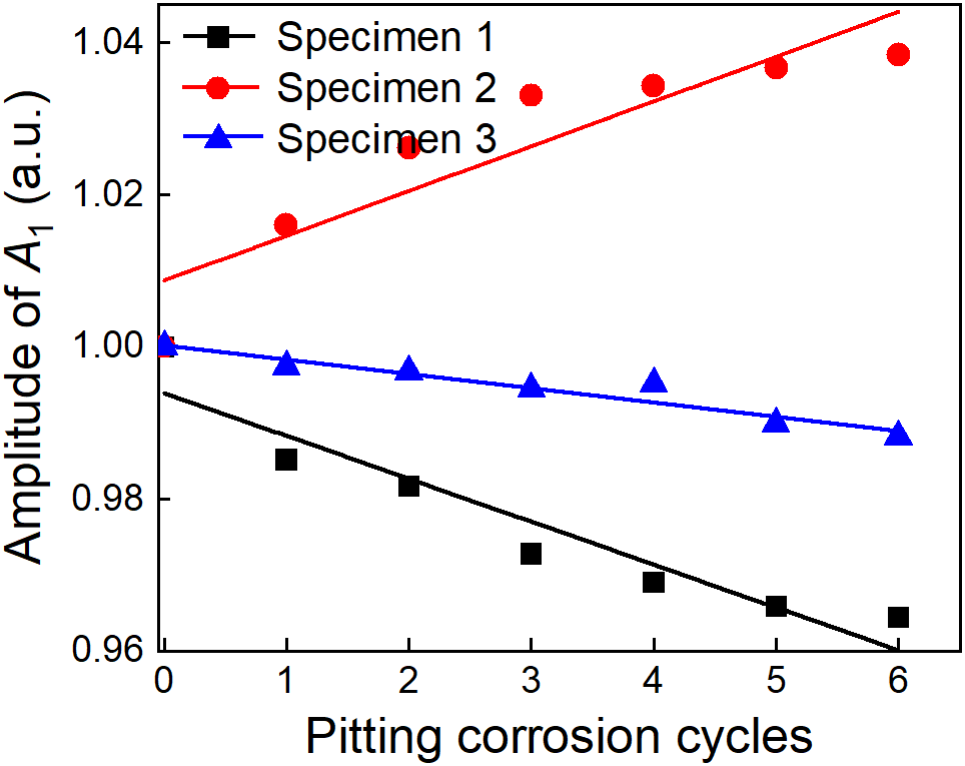
Pitting corrosion cycles versus ultrasonic amplitude in three specimens.

As the number of pitting corrosion cycles increased, the detected ultrasonic signals exhibited similar waveforms and negligible amplitude variations, regardless of the pitting corrosion state. Thus, $A_{\text{1}}$ was selected as the linear damage index because it can represent the fundamental amplitude of the L(0, 2) mode and facilitate comparison with the nonlinear ultrasonic sensitivity. For comparative analysis, all amplitude values were normalized against the initial fundamental amplitude in the intact state. The linear fit is utilized to obtain the slope for sensitivity comparison, rather than implying a strictly linear physical degradation. Consequently, damage accumulation drives a monotonic trend between the damage index and pitting corrosion. The normalized amplitude of the fundamental signal at different number of pitting corrosion cycles for three specimens is shown (Fig 10). The ultrasonic amplitude was fitted with a linear function. The slopes (mean±standard error) of the fitting lines for the three specimens were −0.00564 ± 8.01 × 10^−4^, 0.00589 ± 1.17 × 10^−3^, and −0.00187 ± 2.45 × 10^−4^, with coefficients of determination ($R^{\text{2}}$) of 0.91, 0.83, and 0.92, respectively.

Comparing the slopes across the three specimens revealed inconsistent ultrasonic amplitudes trends, indicating that the linear damage index is highly susceptible to specimen-to-specimen variations. This demonstrates that a high $R^{\text{2}}$ value alone does not guarantee the overall suitability or repeatability of the detection method. Several factors may contribute to this inconsistency. First, the ultrasonic detection system was not sufficiently stable to obtain accurate signal amplitudes. Second, the mode-mixing problem easily occurred because of the dispersed characteristics of the ultrasonic guided wave, causing fluctuations in the amplitude of the fundamental wave. Third, the dimensions of the small-scale craters and microcracks were significantly smaller than the ultrasonic wavelength, decreasing the effect on the transmission signals. Fourth, the variability in $R^{\text{2}}$ and slopes among the specimens can be attributed to the stochastic nature of pitting corrosion. Even after identical corrosion cycles, the specific depth, shape, and spatial distribution of small-scale craters and microcracks vary, leading to different scattering effects on the fundamental waves. Thus, under the specific experimental conditions of this study, linear ultrasonic detection exhibited noticeable limitations in consistently assessing the number of pitting corrosion cycles.

**Fig 11:**
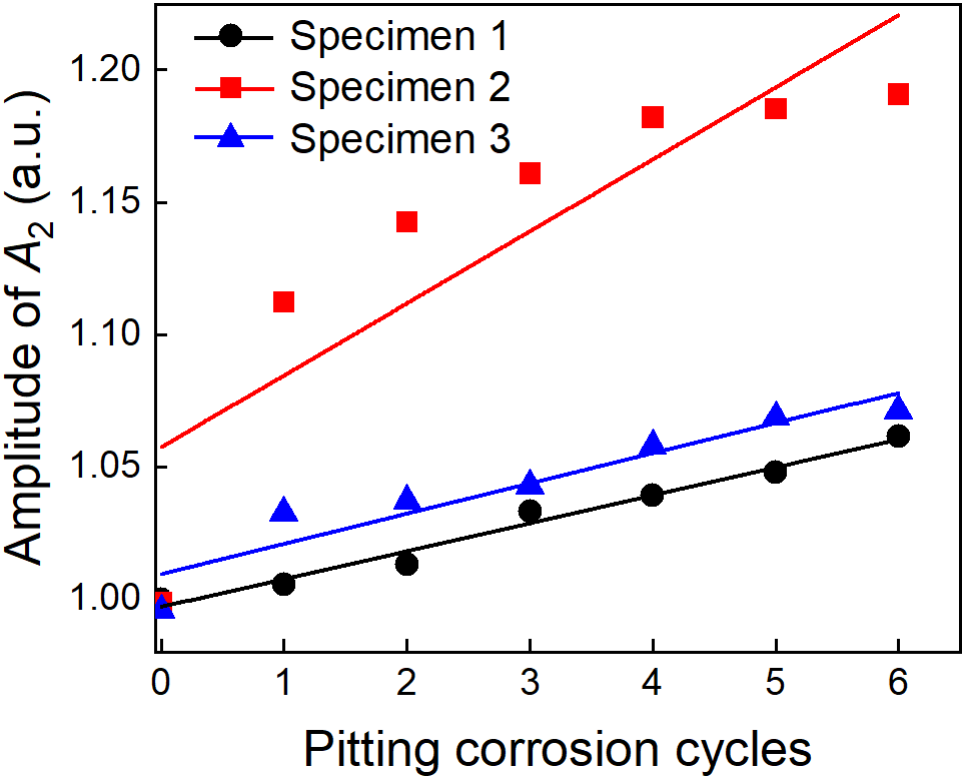
Pitting corrosion cycles versus second-harmonic amplitude in three specimens.

The normalized amplitude of the extracted second-order harmonic wave for different numbers of pitting corrosion cycles is shown (Fig 11). The amplitude of the second-order harmonic wave increased monotonically with the number of pitting corrosion cycles and could be fitted using a linear function. The slopes (mean±standard error) of the fitting lines for the three specimens were 0.01053 ± 6.65 × 10^−4^, 0.02721 ± 6.99 × 10^−3^, and 0.0114 ± 1.71 × 10^−3^, with coefficients of determination ($R^{\text{2}}$) were 0.98, 0.75 and 0.90, respectively. These results indicate the second-harmonic detection exhibits greater regularity than those of linear ultrasonic detection (Fig 10). This monotonic increase in the second-harmonic amplitude can be explained by the wave-damage interaction observed in the micrographs (Fig 4). In the initial pitting corrosion cycles, the small-scale craters induced by corrosion act as local stress concentrations. This induces a nonlinear elastic response where the stress-strain relationship deviates from Hooke’s Law, thereby distorting the ultrasonic guided wave. As the number of pitting corrosion cycles increases, the microcracks highlighted in (Fig 4c) become the dominant source of nonlinearity. Under the alternating stress of the L(0, 2) mode, these microcracks periodically open and close inducing “breathing crack” motion. This contact acoustic nonlinearity (CAN) contributes more significantly to the second-harmonic generation than material nonlinearity. The increasing density and accumulation of these small-scale craters and microcracks (Fig 4a to 4f) drive the monotonic increase in $A_{\text{2}}$.

However, the differences in the variation of the second-harmonic amplitude among the three identical specimens may be attributed to several factors. As demonstrated in the Section Temperature experiments, $A_{\text{2}}$ is highly susceptible to thermal variations. Even minor fluctuations in environment temperature during the long-duration corrosion cycles can change material damping, weaken the adhesive layer, and affect transducer sensitivity, thereby contributing to the observed variance among specimens. In addition to these environmental factors, inconsistencies in experimental operations, equipment instability, and the intrinsic impossibility of achieving a perfectly uniform internal microstructure among the specimens also contribute to the observed differences among the specimens.

**Fig 12:**
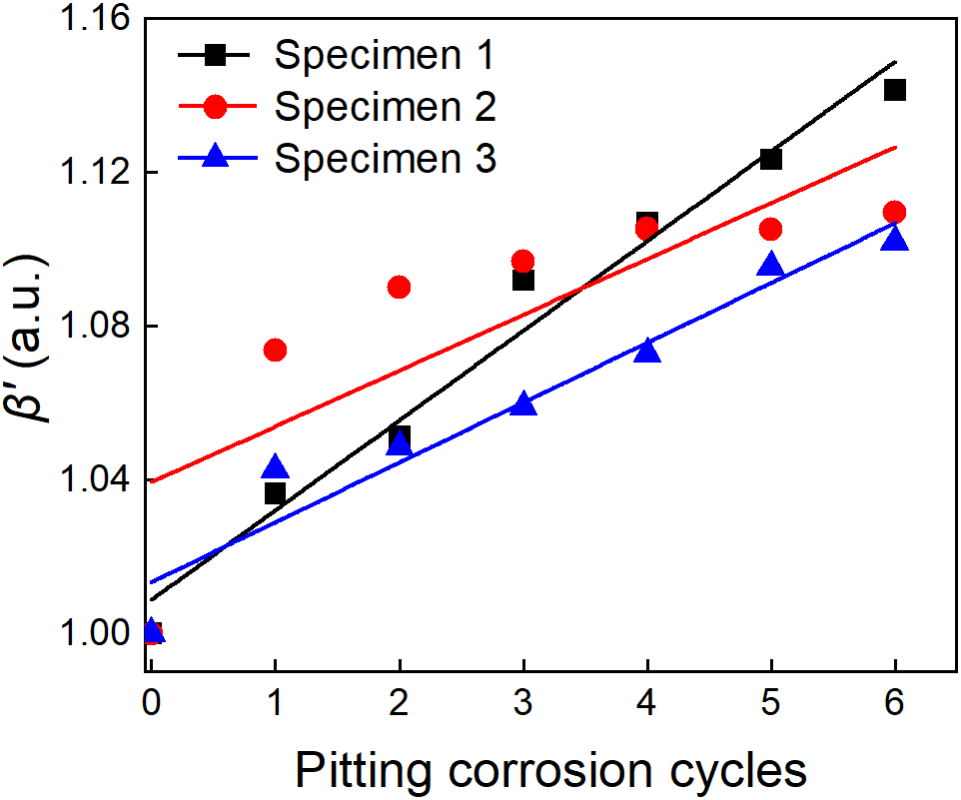
Pitting corrosion cycles versus relative nonlinear parameter $\beta^{\prime }$ for three specimens.

The relative nonlinear parameter $\beta^{\prime }$ as a function of the number of pitting corrosion cycles in the three specimens is shown (Fig 12). Experimental results reveal that the amplitude of $\beta^{\prime }$ increased monotonically over the number of pitting corrosion cycles. The slopes (mean±standard error) for three specimens were 0.02334 ± 1.61 × 10^−3^, 0.01452 ± 4.63 × 10^−3^, and 0.01556 ± 1.76 × 10^−3^, with $R^{\text{2}}$ values of 0.97, 0.66, and 0.94, respectively. The increase in $\beta^{\prime }$ can be explained by the *β*_*CAN*_ induced by crack–wave interactions, as well as the increase in $\beta_{G}$ and $\beta_{M}$ resulting from the growing size and density of cracks and craters. Overall, a monotonic increasing trend is observed across all specimens despite noticeable scatter in the data. Specifically, the $R^{\text{2}}$ value drops to 0.66 in one specimen, creating a noticeable asymmetry compared to the strong goodness-of-fit ($R^{\text{2}}$>0.94) of the other two. This deviation is primarily attributed to the highly stochastic nature of pitting corrosion. As discussed regarding the specimen variability (Fig 10), this substantial specimen-to-specimen variability is primarily attributed to the highly stochastic nature of pitting corrosion damage. The shapes, depths, and spatial distribution of the small-scale craters and microcracks progress randomly in each cycle, leading to localized fluctuations in the nonlinear ultrasonic response. Microstructural differences among pipe specimens and slight inconsistencies in the sensor adhesive layer further contribute to differences among specimens.

Despite the observed data scatter and slope variations, the consistently positive correlation across all experiments supports using $\beta^{\prime }$ to evaluate the accumulation of pitting corrosion damage. The growth rate of $\beta^{\prime }$ was similar to that of the second-harmonic amplitude results for each specimen. Additionally, the goodness-of-fit ($R^{\text{2}}$) of $\beta^{\prime }$ was also similar to that of the linear ultrasonic $A_{\text{1}}$ and second-harmonic waves $A_{\text{2}}$ in the experiments. This finding revealed that the changes in the $A_{\text{2}}$ and $\beta^{\prime }$ may be more suitable for the assessment of corrosion cycles than those in linear ultrasonic detection. Furthermore, the absolute value of $\beta^{\prime }$ may also be influenced by attenuation, dispersion and the frequency-dependent response of the transducers. However, these systematic influences act as constant scaling factors since the experimental configuration remained consistent across all experiments. Additionally, system linearity validation (Fig 8) demonstrates that the inherent nonlinearity of the ultrasonic system is negligible. Therefore, although the absolute value of $\beta^{\prime }$ incorporates baseline system responses, its relative variations effectively reflect the evolution of nonlinearity induced by pitting corrosion.

### Assessment of pitting corrosion area

**Fig 13:**
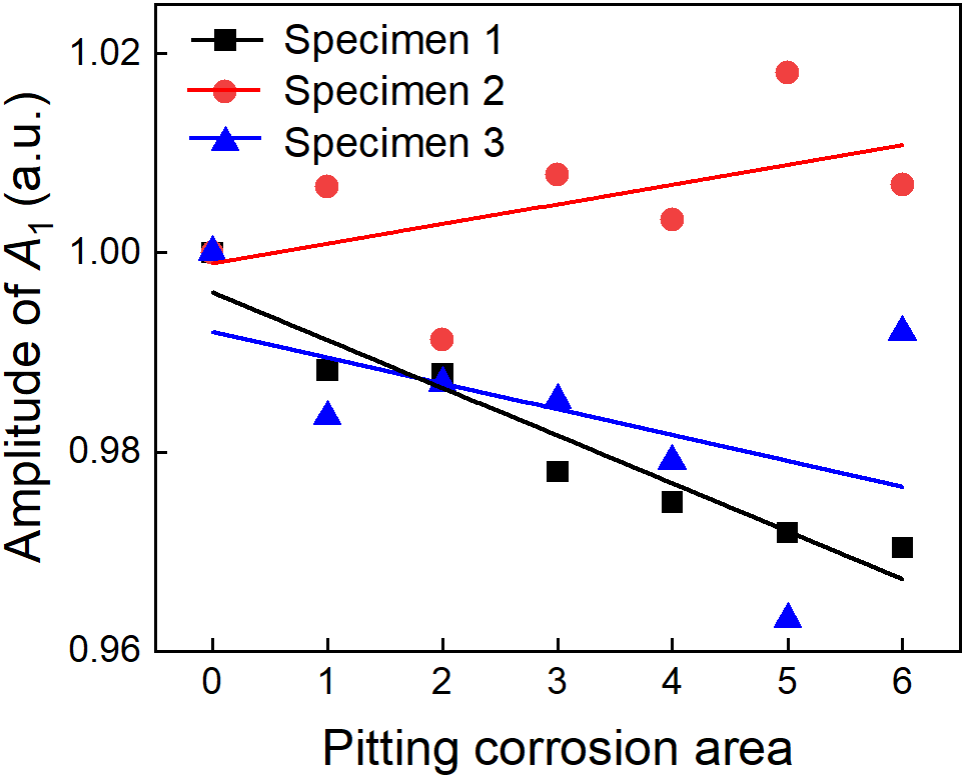
Pitting corrosion area versus ultrasonic amplitude for three specimens.

The normalized amplitudes of the ultrasonic guided waves for the three specimens in different corrosion areas are shown in (Fig 13). The linear fitting slopes (mean±standard error) of the three specimens were −0.00479 ± 6.15 × 10^−4^, 0.00198 ± 1.44 × 10^−3^, and −0.00259 ± 2.07 × 10^−3^, with $R^{\text{2}}$ values of 0.92, 0.27 and 0.24, respectively. These experimental results revealed that the repeatability of the curves was poor owing to the large differences in the slopes among the three specimens. The corrosion-induced small-scale craters and microcracks were too small for accurate detection by linear ultrasonics. The significant variation in $R^{\text{2}}$ values (0.92, 0.27 and 0.24) demonstrates that linear ultrasonic guided waves are easily affected by the random orientations and distributions of small-scale craters and microcracks. Consistent with the results in (Fig 10), the findings in (Fig 13) indicate that the number of cycles and area of pitting corrosion cannot be accurately evaluated using linear ultrasonic amplitudes. Thus, although traditional linear ultrasonic technology shows moderate correlation at certain phases, it exhibits limitations in characterizing the early-stage pitting corrosion and associated microdamage investigated in this study.

**Fig 14:**
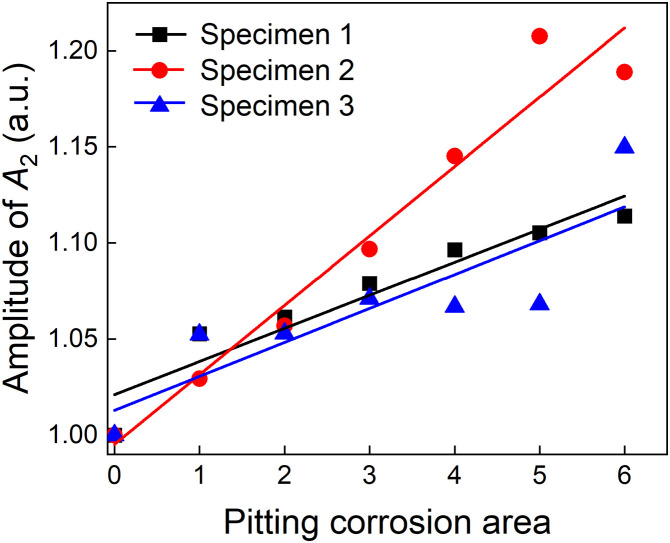
Pitting corrosion area versus second-harmonic amplitude for the three specimens.

The normalized amplitudes of the second-harmonic wave changes in different pitting corrosion areas for the three specimens are shown in (Fig 14). The amplitude of the second-harmonic wave increased monotonically with increasing pitting corrosion area. This aligns with the trend observed in (Fig 11), where the second-harmonic wave similarly varies with the number of pitting corrosion cycles. The slopes (mean±standard error) for the three specimens were 0.01723 ± 2.5 × 10^−3^, 0.03614 ± 3.54 × 10^−3^, and 0.01766 ± 4.64 × 10^−3^, with corresponding $R^{\text{2}}$ values of 0.90, 0.95 and 0.74, respectively. This amplitude increase is attributed to an increase in the number of small-scale craters and microcracks, which enhances the generation of second-harmonic components through ultrasonic guided wave distortion. Although these specimens mostly show early-stage features, the local stress concentration around these small-scale crater edges and microcracks are sufficient to generate the contact acoustic nonlinearity (CAN).

**Fig 15:**
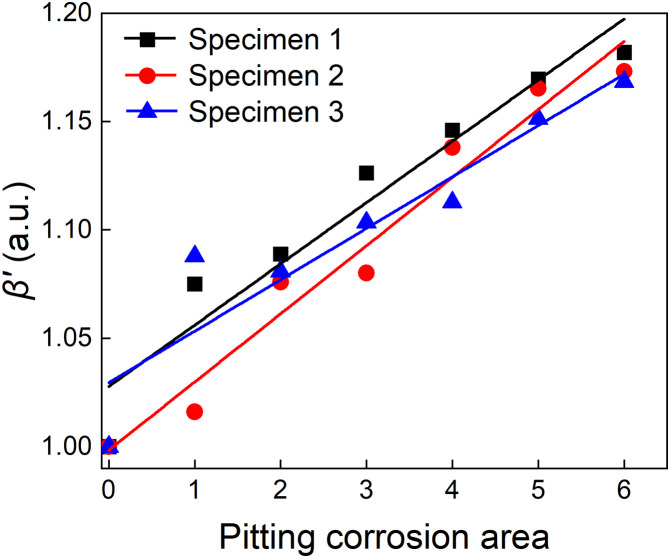
Pitting corrosion area versus relative nonlinear parameter $\beta^{\prime }$ for three specimens.

The regression analysis between the relative nonlinear parameter $\beta^{\prime }$ and the pitting corrosion area for the three specimens is presented in (Fig 15). The β*’* increased monotonically over the pitting corrosion area. The slopes (mean±standard error) for the three specimens were 0.02826 ± 3.39 × 10^−3^, 0.03142 ± 2.74 × 10^−3^, and 0.02371 ± 4 × 10^−3^, with $R^{\text{2}}$ values of 0.93, 0.96 and 0.87, respectively. Given the relatively consistent slopes and high $R^{\text{2}}$ into consideration, it is evident that the growing number of small-scale craters and microcracks increases the ultrasonic nonlinearity contributions from $\beta_{CAN}$, $\beta_{G}$ and $\beta_{M}$, resulting in an increase in $\beta^{\prime }$. The experiments demonstrated that the nonlinear ultrasonic results for the pitting corrosion area were similar to those for the pitting corrosion cycles. Comparing the linear and second-harmonic data reveals that the accuracy and sensitivity of nonlinear ultrasonic detection for pitting corrosion damage are comparatively better than those obtained using the linear ultrasonic method. The nonlinear ultrasonic method can be used to assess changes in the number of pitting corrosion cycles and the area of aluminum pipes. In summary, the pitting corrosion cycle experiments show that progressive accumulation of small-scale craters and microcracks increases the ultrasonic nonlinearity. Similarly, the pitting corrosion area experiments demonstrate that under the present excitation frequency and mode selection, a larger distribution of this damage also increases ultrasonic nonlinearity, as more damage sources contribute to the overall CAN effect.

### Comparative discussion

The performance of $A_{\text{1}}$, $A_{\text{2}}$, and $\beta^{\prime }$ in assessing pitting corrosion cycles and the area is compared in (Table 2). The performance indices, including sensitivity (slopes±standard error), goodness-of-fit ($R^{\text{2}}$), and repeatability (relative standard deviation) are derived from (Figs 10–15). $\beta^{\prime }$ demonstrated comparatively better sensitivity than $A_{\text{1}}$ and $A_{\text{2}}$ due to its highest mean slopes. Moreover, $A_{\text{2}}$ and $\beta^{\prime }$ exhibit high coefficients of determination ($R^{\text{2}}$), especially in pitting corrosion area monitoring, where their $R^{\text{2}}$ values were higher than those of $A_{\text{1}}$. This finding suggests that $A_{\text{2}}$ and $\beta^{\prime }$ may be less affected by systematic instability. Furthermore, $\beta^{\prime }$ had the smallest relative standard deviation across all pitting corrosion experiments, indicating favorable repeatability and stability for pitting corrosion characterization. Thus, despite the minor variability among these specimens, $\beta^{\prime }$ exhibits better stability and normalization capability compared to $A_{\text{1}}$ and $A_{\text{2}}$. However, the repeatability of $\beta^{\prime }$ remains suboptimal, which is likely attributed to variations in the pitting corrosion experiments, fluctuations in system stability, intrinsic differences among specimens, and inconsistencies in MFC sensor bonding. Overall, the results indicate that the sensitivity, goodness-of-fit, and repeatability of nonlinear ultrasonic detection for pitting corrosion cycles are consistent with those for pitting corrosion area.

**Table 2:** Sensitivity, goodness-of-fit, and repetability of nonliner ultrasonic detection.

Index	Pitting corrosion cycles			Pitting corrosion area		
	$A_{1}$	$A_{2}$	$\beta^{\prime }$	$A_{1}$	$A_{2}$	$\beta^{\prime }$
Sensitivity	−0.00054 ± 0.00588	0.01638 ± 0.00939	0.01781 ± 0.00482	−0.0018 ± 0.0035	0.0237 ± 0.0108	0.0278 ± 0.0039
Goodness-of-fit	0.88	0.87	0.85	0.47	0.86	0.92
Repeatability	8.88	0.48	0.23	1.56	0.38	0.12

**Fig 16:**
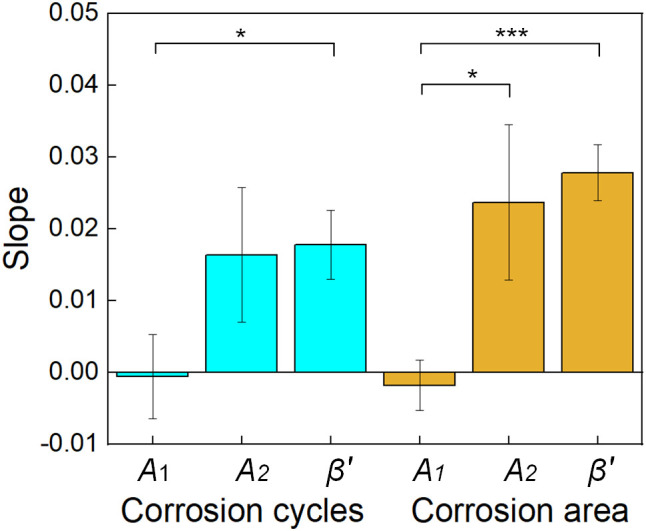
The histogram of slopes and standard error of ultrasonic detection.

A two-sample Welch’s t-test was conducted to investigate the difference in sensitivity slopes between linear and nonlinear ultrasonic detection. This specific method was selected to ensure a robust statistical evaluation, as the limited sample number (three specimens per condition) makes it difficult to justify the assumption of equal variances. The calculated p-values reveal a significant difference between $A_{\text{1}}$ and $\beta^{\prime }$ (Table 3). Particularly for pitting corrosion area detection, the substantial difference in slopes yields a highly significant p-value of 6.3 × 10^−4^ despite the small sample number. This statistical significance is further supported by the error bars (Fig 16). These results indicate that $\beta^{\prime }$ offers better sensitivity and higher detection precision than $A_{\text{1}}$ in characterizing pitting corrosion damage. Therefore, this study demonstrates that nonlinear ultrasonic detection offers more stable sensitivity, goodness-of-fit, and repeatability than conventional linear methods for assessing pitting corrosion in aluminum pipes.

**Table 3:** T-test for different ultrasonic detection.

Variables		(T-test) p-value
Pitting corrosion cycles	$A_{\text{1}}$&$A_{\text{2}}$	0.068
	$A_{\text{1}}$&$\beta^{\prime }$	0.015*
Pitting corrosion area	$A_{\text{1}}$&$A_{\text{2}}$	0.044*
	$A_{\text{1}}$&$\beta^{\prime }$	6.3 **×** 10^−4^***

p < 0.05 statistical difference*, p < 0.01 significant difference**, p < 0.001 highly significant difference***.

## Conclusion

A nonlinear ultrasonic method using MFC transducers was designed to assess the number of cycles and area of pitting corrosion in aluminum pipes based on changes in ultrasonic nonlinearity. Two MFC transducers with flexible properties and favorable ultrasonic performance were used to generate and detect ultrasonic guided waves in pipes with a small outer radius of 10 mm. When the input signal had a frequency of 0.7 MHz, a second-harmonic component of 1.4 MHz was generated and then extracted using the wavelet transform. An experimental study was also conducted to investigate the influence of temperature and system on nonlinear ultrasonic detection. The results indicated that the relative nonlinear parameters are minimally affected by temperatures ranging from 20 °C to 35 °C. The results of the repeatability experiments revealed that the amplitude of the $A_{\text{2}}$ and β′ increase monotonically with the number of pitting corrosion cycles. The slopes for $A_{\text{2}}$ and $\beta^{\prime }$ were 0.01638 and 0.01781, respectively, which were greater than the slope of the linear ultrasonic results (−0.00054). Moreover, the $R^{\text{2}}$ values of the second-harmonic and relative nonlinear parameters $\beta^{\prime }$ were 0.87 and 0.85, respectively. These are similar to the $R^{\text{2}}$ value of the linear ultrasonic amplitude (0.88), indicating a high goodness-of-fit. However, as a high $R^{\text{2}}$ value alone does not fully reflect method reliability. The repeatability and sensitivity were further evaluated. The smaller value of the repeatability index for $\beta^{\prime }$ compared to those of $A_{\text{1}}$ and $A_{\text{2}}$ indicated that $\beta^{\prime }$ has comparatively better repeatability in the detection of pitting corrosion cycles. Nonlinear ultrasonic experiments demonstrated that the amplitudes of $A_{\text{2}}$ and $\beta^{\prime }$ increased monotonically with the pitting corrosion area in the pipes. In addition, $\beta^{\prime }$ exhibited comparatively better sensitivity, goodness-of-fit, and repeatability in pitting corrosion detection compared with other ultrasonic damage index. Therefore, in the present study, observing changes in $\beta^{\prime }$ was found to be more suitable for detecting pitting corrosion than linear ultrasonic and second-harmonic detection results.

These results suggest that nonlinear ultrasonic techniques are effective tools for diagnosing microdamage in various metal structures. Compared to other nondestructive testing techniques, the proposed nonlinear ultrasonic method presents a potential direction for exploring long-distance detection, quick assessment, and real-time online monitoring in future field application. This method offers a promising approach to help address certain measurement limitations of traditional nonlinear ultrasonic methods in detecting localized damage, providing a viable technical solution for the safe operation and maintenance of industrial pipelines. By enabling the timely identification of pitting corrosion, this technology improves detection capabilities and promotes proactive maintenance strategies, ultimately improving asset integrity and operational safety.

The results of the current study also raise several concerns for future research. Future research on nonlinear ultrasonics will expand from the small-sized pipe examined in this paper to pipes of greater length, as well as empty pipes to fluid-filled pipes.

## Supporting information

S1 Data Ultrasonic data under different temperature conditions. (CSV)

S2 Data Ultrasonic data across corrosion cycles. (XLSX)

S3 Data Ultrasonic data and corresponding corrosion areas. (XLSX)

S4 Data Ultrasonic data for system linearity validation. (CSV)
